# Nested Polymerase Chain Reaction and Sequence- Based Detection of *Leishmania* Infection of Sand Flies in Recently Emerged Endemic Focus of Zoonotic Cutaneous Leishmaniasis, Southern Iran

**Published:** 2013-06

**Authors:** Kourosh Azizi, Abdollah Badzohreh, Bahador Sarkari, Mohammad Reza Fakoorziba, Mohsen Kalantari, Mohammad Djaefar Moemenbellah-Fard, Mohsen Ali-Akbarpour

**Affiliations:** 1 Department of Medical Entomology, Research Centre for Health Sciences, School of Health and Nutrition, Shiraz University of Medical Sciences, Shiraz, Iran;; 2 Student Research Committee, Shiraz University of Medical Sciences, Shiraz, Iran;; 3 Department of Parasitology and Mycology, School of Medicine, Shiraz University of Medical Sciences, Shiraz, Iran;; 4 Department of Public Health, Mamasani Paramedical School, Shiraz University of Medical Sciences, Shiraz, Iran;; 5 Department of Diseases Control, Fars Province Health Center, Shiraz University of Medical Sciences, Shiraz, Iran

**Keywords:** Leishmaniasis, PCR, Sand flies, *Phlebotomus papatasi*, *Leishmania major*, Iran

## Abstract

**Background: **Geographical distribution of zoonotic cutaneous leishmaniasis (ZCL) has continuously been extended in recent years in Iran. The Beiza District is one of the newly-emerged endemic foci of ZCL in southern Iran. The main aim of the present study was to detect the vector(s) of ZCL in this area.

**Methods:** To detect the fauna and vectors of ZCL in this district, sand flies were caught using sticky papers. Seventy randomly selected female sand flies out of 730 were molecularly investigated for *Leishmania* infection using species-specific nested polymerase chain reaction** (**PCR) assay between April and October 2010.

**Results:** A total of 2543 sand flies were caught. The fauna was identified as 10 species (five *Phlebotomus* spp. and five *Sergentomyia* spp.). *Phlebotomus papatasi* was the most dominant species both indoors and outdoors (37.55% and 16.35 %, respectively). *L. major* was detected in 5 out of 48 investigated *Phlebotomus* *papatasi* (10.41%). Sequence-based characterization was carried out to confirm the PCR findings. The positive samples were shown to have 75-88% similarity with *L. major *sequences in GenBank.

**Conclusion: **According to the findings of the present study, similar to the other foci of ZCL in Iran, *P. papatasi *is the proven and primary vector of CL. This study could be drawn upon for future strategy planning in this newly emerged endemic focus.

## Introduction

Leishmaniasis represents a wide spectrum of clinical manifestations, including cutaneous (CL), mucocutaneous (MCL), diffused cutaneous (DCL), and visceral (VL) forms. Several variables such as parasite species, vector competence, reservoir hosts, and environmental conditions affect the epidemiology and clinical features of leishmaniasis.^1^^,^^2^

Different species of parasitic protozoan, *Leishmania* (*Kinetoplastida: Trypanosomatidae*), are the causal agents of this vector-borne disease. About 15 *Leishmania* species have been reported to cause leishmaniasis in human. Female phlebotomine sand flies (*Diptera: Psychodidae*) transmit these intracellular parasites to mammals via their infective bites.^3^ Of more than 1000 identified sand flies, thirty species have been reported as proven and 40 as probable or suspected vectors of leishmaniasis.^4^^,^^5^ In Iran, forty-five phlebotomine sand flies have been morphologically identified so far.^6^

Cutaneous leishmaniasis (CL) is endemic in more than 70 countries mainly situated in the tropical and subtropical regions.^3^ More than 90% of CL cases have been reported from Afghanistan, Brazil, Sudan, Iran, Peru, Saudi Arabia, and Syria.^3^^,^^4^ In Iran, CL is endemic in 15 out of the 31 provinces. CL appears in zoonotic and anthroponotic forms, which are caused by *Leishmania* *major* and *L*. *tropica*, transmitted mainly by *Phlebotomus* *papatasi* (Scopoli, 1784) and *P*. *sergenti* (Parrot, 1917), respectively.^7^

The endemic foci of zoonotic cutaneous leishmaniasis (ZCL) are mainly located in three parts of the country, i.e. the central and north-east, west and south-west, and south-east regions.^8^ In all these regions, *P. papatasi* has been reported as the proven and primary vector.^9^^,^^10^ Moreover, some other phlebotomine sand flies have been naturally found infected with *L. major* and considered as probable vectors. These species are *P. *(*Paraphlebotomus*) *mongolensis, P. *(*Phlebotomus*)* salehi, P. *(*Paraphlebotomus*)* caucasicus, P.* (*Synphlebotomus*)* ansarii, and P.* (*Paraphlebotomus*)* alexandri.*^11^^-^^14^

Gerbil rodents (*Muridae: Gerbillinae*) are the reservoir hosts of ZCL in Iran. The main reservoir hosts are *Rhombomys* *opimus*, *Tatera* *indica*, and *Meriones* *hurrianae* in the endemic foci of the three above-mentioned regions, respectively.^15^^,^^16^ Recently, the Baluchistan gerbil, *Gerbillus* *nanus,* has also been naturally found to be infected with *L*. *major* and has been reported as a probable reservoir host in a newly-emerged endemic focus in the south-east parts of Iran.^17^

Nowadays, polymerase chain reaction (PCR)-based assays are routinely used to detect *Leishmania* species in patients, vectors, and reservoir hosts. Nevertheless, the high similarity between the different species of the parasite renders their morphological identification difficult.^8^ To bypass this difficulty, various sources of *Leishmania* DNA, including ribosomal DNA (ssu rRNA), repetitive sequences, kinetoplast DNA (kDNA), and internal transcribed spacer 1 (ITS1), have been used for molecular detection.^13^^,^^18^^,^^19^

Outbreaks of ZCL impose a particularly serious burden of morbidity on people in the rural areas of Iran. The incidence of clinical ZCL cases in the Beiza District, Fars Province, southern Iran, was reported as 16.23 and 12.65/1000 in 2009 and 2010, respectively (Unpublished data, Fars Province Health Center). The present study was aimed to determine the sand fly fauna and detect the potential vectors of *L*. *major* using molecular methods in this new rural focus of ZCL in the Fars Province, south of Iran.

## Methods and Materials

*Study Area*

**Figure 1:**
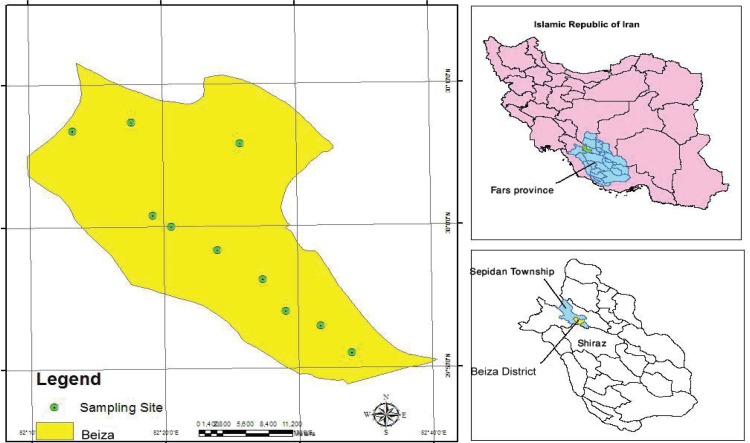
The study area (Beiza District) is located in the Fars Province, south of Iran.

The study was carried out in the Beiza District, Sepidan Township, Fars Province, south of Iran in 2010 (figure 1). This district is situated in a hilly area, south of the Zagross chain of mountains (52°, 10’ E, 29°, 50’ N) with an altitude of 1500-2000 m. The surface area covers 1000 km^2^ with about 30500 inhabitants. The annual average temperature and relative humidity are 14.7°C and 51%, and precipitation is about 52 mm in this county.

*Sand Fly Collection and Species Identification*

Sand flies were collected from 10 villages using sticky papers. Collection of sand flies was carried out twice a month from April to October 2010. Sixty sticky papers were installed per night at each sampling station. The male sand flies were stored in ethanol (70%) for subsequent mounting and species identification. The females were selected for dissection and DNA extraction. 

The head and last abdominal segments were mounted on a microscope slide, in a drop of Puri medium,^20^ so that each sample could be identified in species level, according to the keys given by Lewis, 1982.^21^ The remaining portion of each parous female of the more common Phlebotomus species with no sign of recent blood meal was used for DNA extraction and PCR.

*DNA Extraction*

Total DNA was extracted from each sand fly body, as was described elsewhere.^22^ Briefly, a heat-sealed Pasteur pipette was used to homogenize each body with 200µl of lysis buffer (50 µl Tris-HCl [pH 7.6], 1µl EDTA, and 1% Tween 20) and 12µl of a proteinase K solution (containing 19 µl of the enzyme/ml), in a 1.5-ml microcentrifuge tube. The homogenate was then incubated at 37°C overnight before 300 µl of a phenol: chloroform: isoamyl alcohol mixture (25:24:1, by vol.) were added. After being shaken vigorously, the tube holding the mixture was centrifuged (10,000 xg for 10 min). Thereafter, the DNA in the supernatant solution was precipitated with 400 µl of cold, pure ethanol, resuspended in 50 µl of double-distilled water (DDW), and stored at -20°C until use.

*Amplification of Kinetoplastic Minicircle DNA from Sand Flies*

The nested PCR assay was employed to amplify the kDNA of the* Leishmania* parasites. The assay was carried out in two rounds using the primers of CSB1XR (ATT TTT CGC GAT TTT CGC AGA ACG) and CSB2XF (CGA GTA GCA GAA ACT CCC GTT CA) for the first round and LiR (TCG CAG AAC GCC CCT) and 13Z (ACT GGG GGT TGG TGT AAA ATAG) for the second round.^19^^,^^23^^,^^24^

First, a total reaction mixture (25 µl) was prepared, which contained 5 µl of template DNA, 200 µl of each deoxynucleoside triphosphate (Cinagen, Tehran, Iran), 1.5 µl of MgCl_2_, 1.0 U of Taq polymerase, 50 µl of Tris-HCl (pH 7.6), 10 µl of CSB1XR, and 10 mM of CSB2XF. PCR reaction was set at 94°C for 5 min, followed by 30 cycles, 30 s at 94°C, 1 min at 55°C, and 1.5 min at 72°C, and then a final extension for 7 min at 72°C in a thermocycler (Eppendorf AG; Humbug, Germany). One µl of the first-round products’ dilution (1/9, by vol.) was used as the templates for the second round of PCR. The reaction for the second round was the same as that for the first, except for the volume of the reaction mixture, which was changed to 30 µl, and the use of the primers of 13Z and LiR.^22^ Additionally, 5 µl of the final products were run on 1.5% (V/V) agarose gel marked with ethidium bromide and visualized by ultraviolet trans-illumination. The size of each band was estimated by comparison with the size of the reference strains.

*Leishmania Reference Strains*

Reference strains of *Leishmania infantum* (MCAN/IR/96/Lon 46), *L. major* (MHOM/IR/54/LV 39), and *L. tropica* (MHOM/IR/89/ARD 2) were used as standards. All of these strains were obtained from the Medical Parasitology Laboratory, School of Public Health, and Institute of Health Research, Tehran University of Medical Sciences. Also, DDW was included in each run as a negative control.

*Sequencing*

The PCR products of all the positive samples were purified using the Gel Purification Kit (AccuPrep^®^, Cat. No. k-3035-1, Bioneer, USA). Both forward and reverse sequencing of the strands of amplified DNA were sequenced with the PCR primers on an automated sequencer (Applied Biosystems 377XL). After utilization of the TritrypDB blast program, the nucleotide homologies of the sequenced products were evaluated with *Leishmania* spp., available in GenBank. The determination of sequences was performed using the FASTA formatted sequences, associated with the Chromas program.^15^

## Results

A total of 2543 sand flies, comprised of 730 females and 1813 males, were collected. Of these, 10 phlebotomine species were identified; they belonged to *Phlebotomus* (5 species) and *Sergentomyia* (5 species).The most prevalent species was *P. papatasi*, representing 53.9% of the total sand flies. This species was the most common species both outdoors and indoors, representing 37.55% and 16.35% of the specimens, respectively. Two species of *Sergentomyia* (*S. baghdadis* and *S*. *squamipleuris*) were just captured outdoors (table 1).

**Table 1:** The species and numbers of male (♂) and female (♀) sand flies caught indoors and outdoors, Beiza District, 2010

**Species**	**Sex**				**Trap**				**Total**	
	**♀**		**♂**		**Out doors**		**In doors**		**N**	**%**
	**N**	**%**	**N**	**%**	**N**	**%**	**N**	**%**		
*P. papatasi*	386	28.15	985	71.85	955	69.65	416	30.35	1371	53.9
*P. sergenti*	211	47	238	53	250	55.67	199	44.33	449	17.6
*P .tobbi*	14	3.7	367	96.3	347	91.07	34	8.93	381	15
*P. salehi*	0	0	38	100	32	84.21	6	15.79	38	1.5
*P. caucasicus*	0	0	31	100	21	67.74	10	32.26	31	1.2
*S. theodori*	102	49.5	104	50.5	151	73.3	55	26.7	206	8.1
*S .clydei*	0	0	20	100	4	20	16	80	20	0.78
*S. dentata*	0	0	30	100	24	80	6	20	30	1.17
*S. baghdadis*	9	100	0	0	9	100	0	0	9	0.35
*S. squamipleuris*	8	100	0	0	8	100	0	0	8	0.3
Total	730	28.7	1813	71.3	1801	70.82	742	29.18	2543	100

Randomly, 70 female specimens, consisting of 48 *P. papatasi*, 17 *P. sergenti*, and 5 *P. tobbi*, were assessed for *Leishmania* infection. *Leishmania* DNA was detected only in 5 (10.41%) specimens of *P. papatasi*, all of which had been collected outdoors from near the rodents’ burrows.

**Figure 2:**
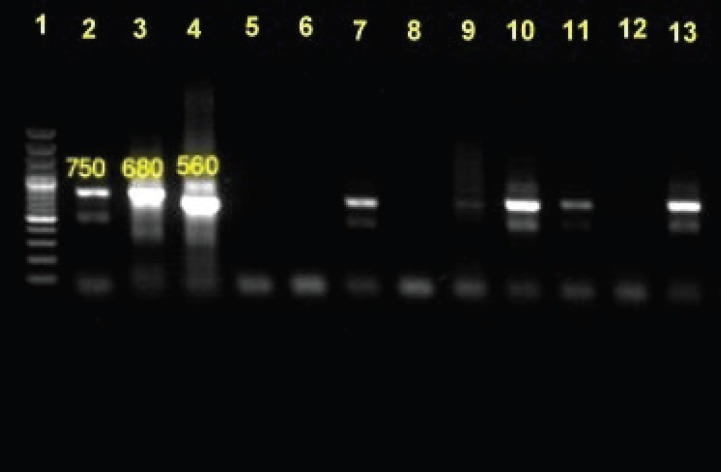
This is an illustration of the results of the polymerase chain reaction-based amplification of kinetoplast DNA. The samples investigated came from 7 wild-caught Phlebotomus papatasi (lanes 7-13) or reference strains of Leishmania tropica (lane 2), L. infantum (lane 3), and L. major (lane 4). Lane1 contained the marker. Lanes 5 and 6 were used as negative controls (double-distilled water).

The band size of the provided impression smears from the *P. papatasi* specimens was about 560 bp, equal to the band size of the *L. major* standard strain. No amplicon was detected in the band size of *L. tropica* (750 bp) and negative samples (table 2, figure 2).

**Table 2:** Number and percentage of the infected dominant phlebotomine sand flies, Beiza District, Fars Province, 2010

**Species**	**Total Females**	**Investigated**		**Found Infected**	
		**N**	**%**	**N**	**%**
*P. papatasi*	386	48	12.43	5	10.41
*P. sergenti*	211	17	8.05	0	0
*P. tobbi*	14	5	35.71	0	0
Total	611	70	1145	5	10.41

Using TritrypDB sequence analysis against the *Trypanosomatidae* species, the target sequence of the PCR products showed 75-88% similarity with *L. major*. All the positive samples demonstrated 75-88% similarity with *Leishmania major* Friedlin Lm-FR-18 kinetoplast minicircle (GenBank: EU370903.1) and *Leishmania major* isolate Lm-FR-9 kinetoplast minicircle (GenBank: EU370908.1).

## Discussion

Leishmaniasis occurs as a spectrum of clinical syndromes. With a broad differential diagnosis courtesy of its diverse clinical presentations, CL is a zoonotic disease with a wide range of mammalian reservoirs and vectors. It may be widespread on a global scale or focal at a local level because of the specific habitat requirements of the sand fly vectors and its various reservoir hosts.^11^

Our data showed that *P. papatasi* was the most dominant species both indoors and outdoors. This species has been adapted to live in human and domestic animal shelters and is largely found in habitats such as bricks and clay houses, stables, and other man-made structures.^25^ *P. papatasi* is widespread in the semi-arid and arid regions of Mediterranean Europe, North Africa, Middle East, and the Indian subcontinent.^25^^-^^27^ All of the 10 identified species in this entomological survey have been previously found in southern Iran.^22^^,^^28^

The epidemiology of ZCL varies based on the bio-ecology of the vectors and the species of the species;^25^ as a result, a necessary factor for designing any effective control strategy is the detection of vectors and their biology.^22^

Molecular techniques based on parasites’ DNA are useful for this purpose and have been commonly used worldwide.^29^^,^^30^ The nested PCR assay is more sensitive than microscopic dissection for identifying *Leishmania* infection in sand flies.^9^ In the Rodrigues *et al.* study,^31^ the PCR specific for the subgenus *Viannia *had a sensitivity of 95.4%, whereas the genus-specific PCR detected the target DNA in 88.2% of the subgenus *Leishmania* samples tested. The specificity of the PCR assay, determined with samples from a group with nonleishmaniasis CL, was 100%.^31^

This is the first report of natural infection of *P. papatasi* with *L*. *major* in this endemic focus of ZCL. *L. major* DNA was detected in 5 (10.41%) specimens of *P. papatasi*. In recent years, most molecular studies carried out in different parts of Iran, e.g. the Isfahan and Fars Provinces (two important endemic foci of ZCL in the centre and south of Iran), have shown that *P. papatasi* has a key role in *L. major* transmission.^8^^-^^10^ Davami et al.^32^ in a similar study based on observation of amastigotes in dissected sand flies, used a high-sensitive and specific nested-PCR assay designed for kDNA of Leishmania in order to compare the kDNA of sequenced products with GenBank. The results confirming the highest homology of greater than 75% with *L. major*, the authors concluded that the species isolated from the sand flies was *L. major*.^32^

All the infected *P. papatasi* in this study were parous and caught at the entrance to gerbil burrows and from the homes of cases with CL. The PCR assay cannot differentiate between the amastigotes (parasite form of macrophage cells in reservoirs) and promastigotes (infective parasite form in vectors) from a blood-meal infected sand fly.^33^ Consequently, in this study, we used parous specimens, whose swallowed amastigotes had converted to promastigotes during blood digestion in the alimentary gut and whose parasite life cycle had partially completed. The detected DNA in this survey might, therefore, belong to the promastigote form.

## Conclusion

According to the findings of the present study, *P. papatasi* is reported as a proven/primary vector of *L. major* in the Beiza District based on its high abundance and natural infection. 
